# In-Loco Optical Spectroscopy through a Multiple Digital Lock-In on a Linear Charge-Coupled Device (CCD) Array

**DOI:** 10.3390/s23167195

**Published:** 2023-08-16

**Authors:** Hugo Fonsêca, Diego Rativa, Ricardo Lima

**Affiliations:** 1 Department of Computer Engineering, University of Pernambuco, Recife 50720-001, Brazil; haf@poli.br (H.F.); diego.rativa@poli.br (D.R.); 2 Department of Systems Engineering, University of Pernambuco, Recife 50720-001, Brazil

**Keywords:** CCD spectrometer, signal processing, digital lock-in amplifier

## Abstract

Accurate and reliable measurements of optical properties are crucial for a wide range of industrial and commercial applications. However, external illumination fluctuations can often make these measurements challenging to obtain. This work proposes a new technique based on digital lock-in processing that enables the use of CCD spectrometers in optical spectroscopy applications, even in uncontrolled lighting conditions. This approach leverages digital lock-in processing, performed on each pixel of the spectrometer’s CCD simultaneously, to mitigate the impact of external optical interferences. The effectiveness of this method is demonstrated by testing and recovering the spectrum of a yellow LED subjected to other light sources in outdoor conditions, corresponding to a Signal-to-Noise Ratio of −70.45 dB. Additionally, it was possible to demonstrate the method’s applicability for the spectroscopic analysis of gold nanoparticles in outdoor conditions. These results suggest that the proposed technique can be helpful for a wide range of optical measurement techniques, even in challenging lighting conditions.

## 1. Introduction

Several optical techniques are used to analyze the interaction between light and matter in visible and infrared spectrums, providing valuable information about their optical and structural properties [1,2,3]. By studying the reflectance, scattering, transmittance, absorbance, and fluorescence of light intensity spectrums, materials can be characterized more accurately [4,5,6,7,8]. As such, a spectrometer is an indispensable tool for performing these experiments. In modern instruments, diffraction gratings are employed to separate polychromatic light into each wavelength using constructive and destructive interference. Monochromators also use this principle; however, they have a rotating base that allows them to select a specific wavelength that passes through an exit slit [4,5,9,10].

In order to ensure accurate readings, spectroscopy setups are usually limited to laboratories with well-controlled illumination, which is necessary to manage the Signal-to-Noise Ratio (SNR) and reduce external optical interference, such as light from surrounding media or its variations. Miniaturized and portable spectrometers have been adopted in most lab research, which utilizes a fixed grating that disperses light onto a CCD array sensor. The spectral resolution of this setup is limited by the pixel size and separation, with digital intensity values transmitted via USB protocols [9,10]. By limiting these setups to controlled environments, it becomes possible for researchers to fully exploit their potential capabilities and use them for industrial applications.

Portable spectrometers are broadly explored in several productive sectors, such as the chemical [11] and mechanical sectors [12] and in agriculture [13,14,15,16]. For instance, Felix Instruments Company has developed types of equipment to perform in-loco near-infrared spectroscopy (λ = 640–1050 nm), analyzing the fruit quality based on light reflectance [17]. The device has a simple and clever optical design that explores the fruit as a light block, avoiding external optical interferences. However, that idea does not apply to other optical techniques, such as scattering or absorption characterization setups.

Optical signals often have low SNR or are measured with electronic systems that introduce noise. To enhance the SNR, well-established techniques such as lock-in or synchronous amplifiers can be used. These techniques are based on homodyne detection and low-pass filtering and allow for the extraction of information from signals with low SNR by comparing the amplitude and phase of the signal to a periodic reference signal [18,19,20]. The concept of phase-sensitive detection has been improved by implementing a two-phase blocking system, which enables vector tracking [21]. The lock-in device is present in different research laboratories with different purposes, such as the characterization of electrical impedance [22,23,24,25,26,27,28,29], biosensor [30,31,32] and optical spectroscopy [33,34,35,36,37,38,39]. The development of portable lock-in amplifiers are carried out via analog circuits, microcontrollers, FPGA, or a combination of these technologies, with SNR results between −24 and −57.5 dB [40,41].

The lock-in technique has also been successfully implemented with computational techniques and digital processing via software, enabling multiple lock-in processings with multi-spectral and multi-frequency applications [40,42,43,44,45]. Unlike analog processing, the computational domain avoids noises due to thermal fluctuation, a better suppression of harmonics, more flexible processing options, and greater precision [46]. The SNR commonly found in a computer lock-in amplifier is around −32 dB [40,47].

This paper proposes a novel approach that enables the exploration of CCD spectrometers for optical spectroscopy applications without the need for external luminosity control through computational techniques. The Lock-In Linear CCD Array Methods section described the lock-in processing of the information obtained from the CCD spectrometer, as well as the development of a program called SpectroPlot using C#, which includes the Detection and Modulation modules.

These modules have been designed to validate the methodology. In the Results and Discussion section, we present the results of optical experiments involving the spectral recovery of a yellow LED and the absorption of gold nanoparticles. The method accurately identifies the absorption spectra of the gold nanoparticles, even under the influence of solar radiation and correlation. The method has been successfully tested in outdoor scenarios, specifically under typical cloudy day conditions where variations in solar radiation fluctuation of 65% are expected. This method is proved in the outdoor tests under typical cloudy day condition where variations in solar radiation fluctuation of 65% are expected. This versatile method can be applied in various environments with diverse lighting conditions, including solar radiation. It proves to be useful in on-site analysis systems based on optical techniques, such as diffraction, scattering, fluorescence, microscopy, and polarimetry, among others.

## 2. Lock-In Linear CCD Array Methods

**Figure 1:**
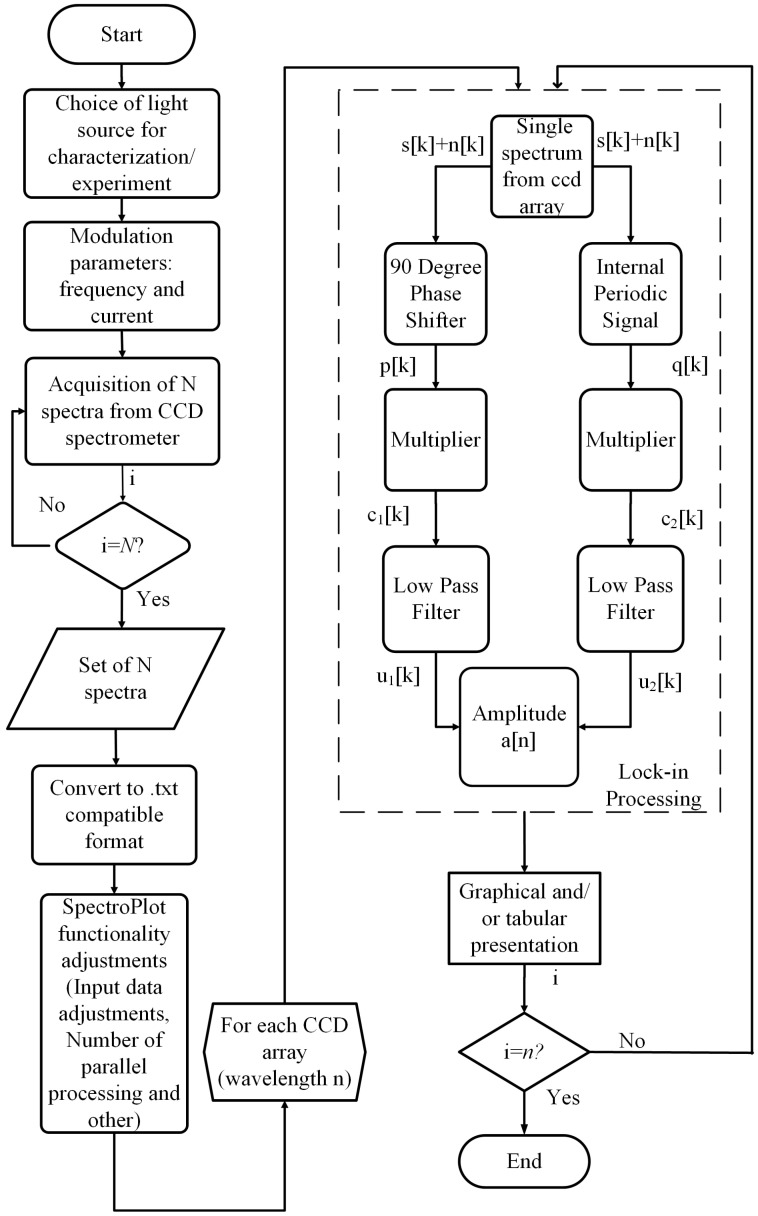
Block diagram of the proposed methodology.

The flowchart in Figure 1 illustrates the proposed approach for implementing the lock-in linear array. Initially, the user specifies the light source for either characterization or an optical spectroscopy experiment. Depending on the modulation parameters, the lamp’s light source can be modulated using external optical choppers that can be digitally triggered. Alternatively, LEDs and diode lasers can be modulated directly from their bias currents through microcontroller platforms, which adjust the modulation current and frequency.

The raw data from the CCD spectrometer can be saved and organized in a file format suitable for analysis. Typically, the spectrometer’s proprietary software allows users to capture *N* spectra during a specified duration, with each spectrum depicted as a series of data points corresponding to the number of pixels on the sensor. The recorded data can be converted into standard file formats, such as .txt or .csv, to facilitate further analysis. The acquisition speed of the spectrometer is influenced by factors such as the user-set exposure time and the internal shutter speed.

**Figure 2:**
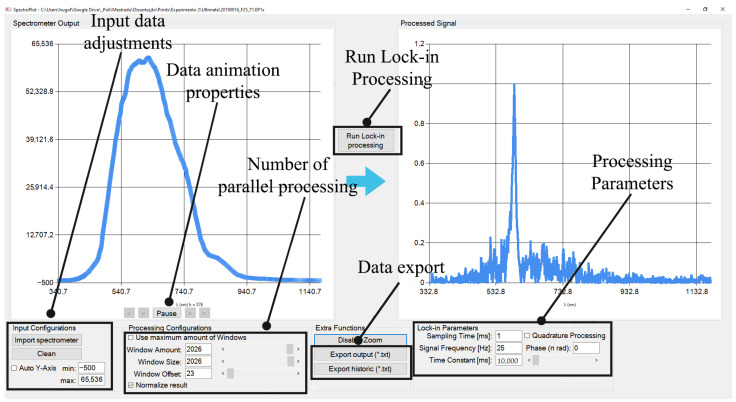
Graphical interface of the software.

The SpectroPlot program, written in the C# programming language, has been created to handle the spectral data. This program has several features, including an animated graphical display of the input and output spectra, user-adjustable input data, and an option for output normalization. Additionally, SpectroPlot enables multiple lock-in processing, a checkbox for quadrature processing (phase = 90°), the generation of text files with intermediate steps and final results, and the adjustment of lock-in parameters such as sampling, time constant, reference signal modulation, frequency, and phase (for non-quadrature processing). Figure 2 depicts the graphical interface of SpectroPlot, which includes the following functionalities:

- Input data adjustments. For the Y-axis visualization properties (setting minimum and maximum Y-axis values), the spectrometer data file (*.EP1x*) is imported, and all current files loaded are cleared.
- Data animation properties. The spectrometer input is 3-dimensional data: wavelength, amplitude, and time. Consequently, the input data are shown as animations, where the horizontal axis is the wavelength, the vertical axis is the amplitude, and each frame represents a step in time.
- The number of parallel processing fields containing the amount of simultaneous lock-in configuration and the width of the processing window are determined. The user can process a specific wavelength instead of the whole spectrum.
- The lock-in processing button that starts the functionality of lock-in processing is run, merging the lock-in parameters and the input data.
- Data export functionality allows the user to either export the final result of the lock-in processing (export output). It can be a two-dimensional (wavelength and normalized amplitude) text file or each intermediary step of the lock-in processing as a 3-dimensional text file (time, wavelength, and normalized amplitude).
- Processing parameters. The user can adjust all usual lock-in parameters, such as sampling period, time constant, reference signal modulation frequency, and phase (in case of no quadrature processing), and check a checkbox for quadrature processing (phase = 90°).

The process flowchart depicted in Figure 1 involves the subsequent stage of lock-in processing for each CCD spectrum array. The input of the lock-in system in the discrete-time domain x[k] comprises the signal to be measured s[k] and the noise component n[k] of the CCD sensor pixel, i.e., x[k]=s[k]+n[k]. The modulation signal (square wave) is represented as p[k]. The quadrature reference component q[k] can be generated via software by introducing an internal 90° phase shift. Importantly, synchronization is established between the reference signal generated via the microcontroller platform and the program’s time-counting routines. Therefore, the correlation between the in-phase ($c_{1}\left[k\right]$) and quadrature ($c_{2}\left[k\right]$) components can be expressed as follows:

(1) $$c_{1}\left[k\right]=(s[k]+n[k])\cdot p\left[k\right],$$

(2) $$c_{2}\left[k\right]=(s[k]+n[k])\cdot q\left[k\right],$$

To obtain the lock-in processing outputs, namely $u_{1}\left[k\right]$ and $u_{2}\left[k\right]$, a first-order low-pass filter is applied to the input signals $c_{1}\left[k\right]$ and $c_{2}\left[k\right]$, respectively. By modeling the filter with specific time constant τ and sampling period $T_{s}$, we shape the filter’s behavior in the time domain. The first-order low-pass filter equations for $u_{1}\left[k\right]$ and $u_{2}\left[k\right]$ can be expressed as recursive relationships, where the current output u[k] depends on the previous output u[k−1] and the current input c[k]. The equations for $u_{1}\left[k\right]$ and $u_{2}\left[k\right]$ are as follows:

(3) $$u_{1}\left[k\right]\;=\;\alpha \cdot u_{1}\left[k-1\right]+\left(1-\alpha \right)\cdot c_{1}\left[k\right]$$

(4) $$u_{2}\left[k\right]\;=\;\alpha \cdot u_{2}\left[k-1\right]+\left(1-\alpha \right)\cdot c_{2}\left[k\right]$$

where α is a coefficient that determines the filter’s behavior. To ensure that the filter achieves the desired time constant τ and sampling period $T_{s}$, the value of α is given via the following:

(5) $$\alpha \;=\;e^{-\frac{T_{s}}{\tau }}$$

This value of α ensures that the filter behaves as a first-order low-pass filter with the desired time constant τ and sampling period $T_{s}$. Substituting the value of α into the filter equations, we obtain the following:

(6) $$u_{1}\left[k\right]=e^{-\frac{T_{s}}{\tau }}u_{1}\left[k-1\right]+\left(1-e^{-\frac{T_{s}}{\tau }}\right)\cdot c_{1}\left[k\right]$$

(7) $$u_{2}\left[k\right]=e^{-\frac{T_{s}}{\tau }}u_{2}\left[k-1\right]+\left(1-e^{-\frac{T_{s}}{\tau }}\right)\cdot c_{2}\left[k\right]$$

After a total of *N* successive samples, the amplitude a[ω] for each wavelength ω associated with a single CCD sensor element can be determined. This is achieved by performing successive recursive filtering on the lock-in processing outputs $u_{1}\left[k\right]$ and $u_{2}\left[k\right]$, as described earlier. The values $u_{1}^{2}\left[N\right]$ and $u_{2}^{2}\left[N\right]$ represent the squared magnitudes of the filtered signals after *N* samples. The amplitude a[ω] is then calculated by taking the square root of the sum of these squared magnitudes:

(8) $$a\left[\omega \right]\;=\;\sqrt{u_{1}^{2}\left[N\right]+u_{2}^{2}\left[N\right]}$$

The SpectroPlot processing is accomplished in a computer with an Intel^®^ Core (TM) i5-3337u CPU, 1.8 GHz frequency, and 6 GB RAM, resulting in execution during 100 ms. It is estimated that single board mini computers with 4 1.5 GHz core processors and 4 GB RAM will take around 200 ms to process such results. This processing time indicates that the program requires low computational effort and is aligned with industry 4.0 technology, encompassing the main technological innovations in automation, control, and information technology applied to manufacturing processes. This makes it possible to use SpectroPlot on OEM computer platforms [48].

### Experimental Setup

**Figure 3:**
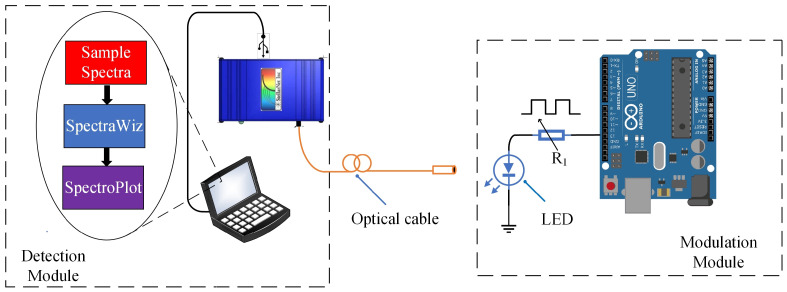
Diagram of the detection and the modulation modules.

Figure 3 illustrates the detection and modulation modules used in this paper. The Detection Module consists of the StellarNet^®^ BLUE -Wave Miniature Spectrometer (StellarNet, EUA, Tampa, FL, USA) (200–1150 nm wavelength range and 2048 element CCD sensor) and a notebook, as shown in Figure 3. The StellarNet obtains the optical spectrum through the SpectraWiz software of the manufacturer. The functionality SpectraWiz for exporting data is called Episodic Data Capture, with a maximum sampling rate of 1 ms, corresponding to 100 spectra captured in 1 s. The exported data is stored in its own encrypted format *.EP1* or in a plain text format (*.EP1x*), posteriorly used by SpectroPlot to perform the lock-in processing.

The Modulation Module consists of an Arduino Uno^®^ microcontroller platform, which is programmed to perform the voltage modulation of the LED from a square wave from a Pulse Width Modulation (PWM) signal (5–0 V). Typically, a semiconductor LED allows PWM modulation in hundreds of kHz. The rate of the spectrum capture data is limited to 1 ms, not allowing the processing of signals with modulations higher than 1 kHz. Keeping this in mind, we set a maximum frequency PWM signal of 25 Hz. It is important to note that lock-in systems generally do not use this frequency range as it increases the system’s susceptibility to noise [18,37,38].

## 3. Results and Discussion

**Figure 4:**
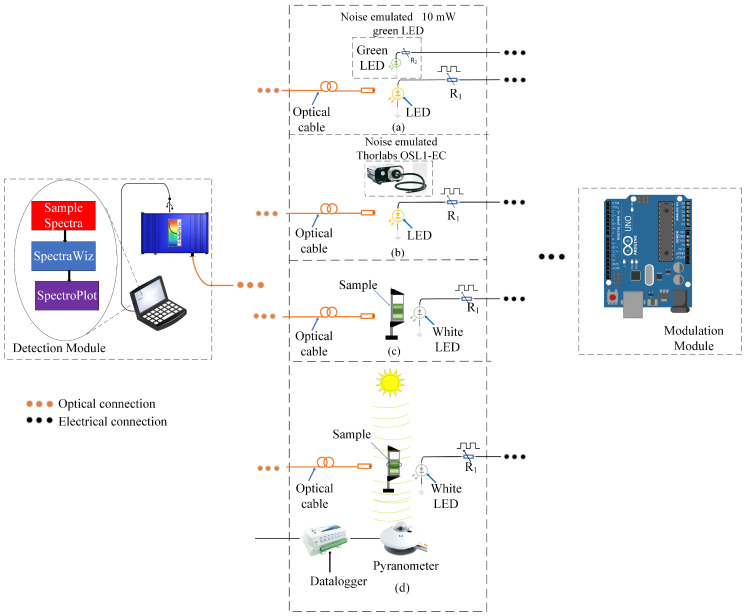
Diagram of the experiments performed. Validation experiments in (**a**) external green light source (**b**) external white light source, (**c**) outdoor absorption spectrum analysis, and (**d**) outdoor correlation analysis.

In this section, four experiments are presented to verify the effectiveness of the lock-in multiple processing methodology in a CCD spectrometer. The two initial experiments, called Validation Experiments, were performed to verify the ability of the method to filter the modulated signal (yellow LED) from other signals. The first experiment explores a green LED containing spectral components that coincide partially with the yellow LED; the second uses a broad-spectrum high-intensity fiber light source. The intention of using the same yellow LED in both experiments stands out because, in this way, it becomes possible to reliably analyze the capacity of the proposed lock-in processing in situations where the modulated LED is subjected to the emulated noise with different spectral contents and intensity. To avoid the pixel saturation of the spectrometer’s CCD matrix, the light sources emulate the noise at a radial distance of 5 cm from the optical fiber. In a third experiment, the absorption spectrum of gold nanoparticles is obtained under external conditions with high fluctuations in solar radiation. Finally, the feasibility of the methodology under external conditions is analyzed, verifying the impact of fluctuations in solar radiation on the detected modulated signal. For these experiments, a 1 W white LED is explored because the spectral content of the white LED encompasses the region of the maximum absorption of the gold nanoparticle under investigation. The first three experiments were performed using the lock-in processing method with the same set of parameters, analyzing a spectrum with 2050 points. These parameters included a sampling time of 1 ms, a time constant of 10 s, and a frequency signal of 25 Hz. However, in the last experiment, the frequency signal of 1 Hz was used. Figure 4 shows the block diagram of the performed experiments.

### 3.1. Validation Experiments

#### 3.1.1. External Green LED Light Source

**Figure 5:**
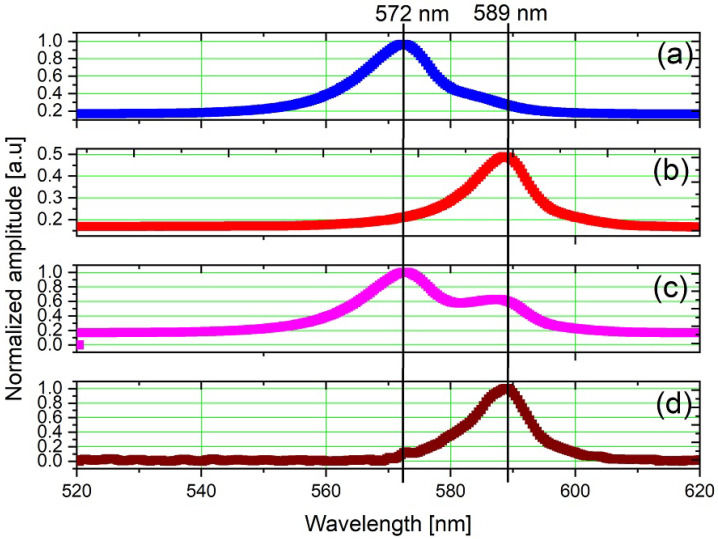
Optical spectrum: (**a**) green LED, (**b**) yellow LED, (**c**) both LEDs on simultaneously, and (**d**) after lock-in processing for the yellow LED subjected to noise emulated by a higher intensity green LED.

Figure 4a shows the experimental setup composed of a yellow LED (λ = 589 nm), modulated in voltage with a PWM signal of 25 Hz and duty cycle of 50%, in an environment containing a green LED (emulate noise), with an emission peak at λ = 572 nm and spectral width of Δλ = 20 nm. The spectrum of the individual and overlapped LEDs can be seen in Figure 5a,b, respectively. The green LED spectrum corresponds to an emulated noise with frequency components that overlap part of the spectral width of the yellow LED, with an SNR of −5.9 dB. The intensity normalization considers the peak value at λ = 572 nm.

The recovered signal displays an emission profile similar to the yellow LED, with a maximum amplitude at the central wavelength of λ = 589 nm and a spectral width of 20 nm, as presented in Figure 5c. The normalization constant is obtained from the maximum intensity values of the yellow LED after lock-in processing. The signal intensity at λ = 572 nm is reduced by approximately eight times, resulting in an SNR of around 19.6 dB after lock-in processing.

#### 3.1.2. External White Noise

**Figure 6:**
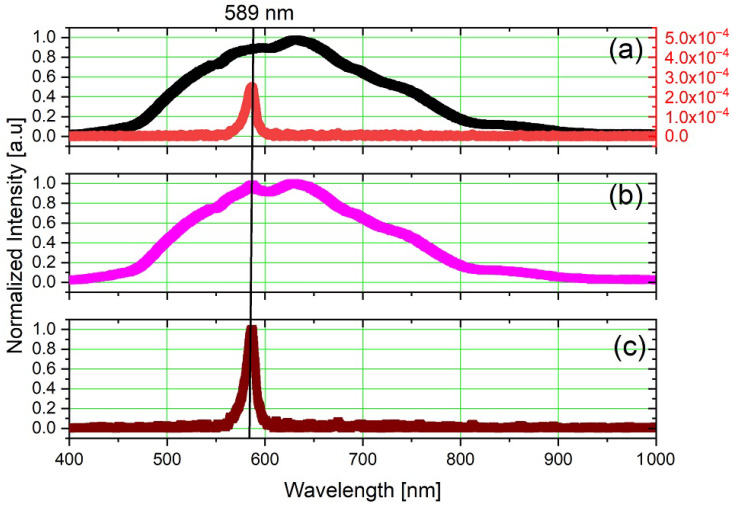
Optical spectrum: (**a**) high-intensity white light and yellow LED separated, (**b**) high-intensity white light and yellow LED simultaneous, and (**c**) after lock-in processing for the yellow LED subjected to noise emulated by high-intensity white light.

Figure 4b shows the experimental setup composed of a modulated yellow LED (λ = 589 nm), modulated in voltage with a PWM signal of 25 Hz and duty cycle of 50%, in an environment with a Thorlabs^®^ OSL1-EC high-intensity fiber light source. The spectrum of the individual and overlapped light sources are shown in Figure 6a,b, respectively. The OSL1-EC acted as the white noise, encompassing the whole spectrum of the yellow LED, as highlighted in Figure 6b, and yielding an SNR of −70.45 dB. The intensity normalization considered the maximum peak value of the external white light source, which occurred at λ = 640 nm, where the power density of the External White Light Source was 3.5 × $10^{3}$ more significant than the power density of the yellow LED.

As shown in Figure 6c, using the proposed method, the yellow LED signal is perfectly recovered, with a maximum amplitude at the central wavelength of λ = 589 nm and spectral width of Δλ = 11 nm.

### 3.2. Outdoor Absorption Spectrum Analysis

The absorption spectrum of an aqueous colloid composed of inclusions of 40 nm gold NanoParticles (NP) subjected to the influence of fluctuations in solar radiation is obtained using a broad light source (1 W white LED), modulated in voltage with a square wave of 25 Hz, as shown in Figure 4c. A Gold NP colloid with a plasmon resonance peak in the visible region (λ=530 nm) [49,50] has been explored, with a volumetric concentration of 0.001%, in a cuvette with an optical length of 1 cm.

**Figure 7:**
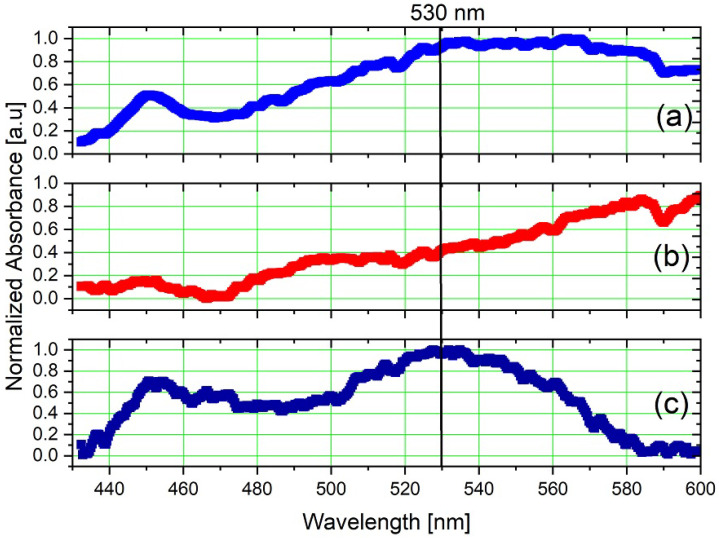
Results of the optical spectroscopy experiment in outdoor colloidal analysis for the (**a**) water only, (**b**) gold colloidal, and (**c**) after lock-in processing.

According to the Beer–Lambert law, the absorbance A(λ), which represents the fraction of the light intensity absorbed by the sample, is given via the following [51]:

(9) $$A\left(\lambda \right)=-\ln\left(\frac{I_{0}}{I(\lambda )}\right)=\sigma \left(\lambda \right)L,$$

where σ(λ) represents the absorption coefficient of the medium and *L* the optical path equivalent to the cuvette’s length. The experiment aimed to measure the absorption spectrum of gold nanoparticles (NP) in a colloid (water) under solar radiation. Firstly, the absorption spectrum of water was obtained as a reference signal, followed by measuring the spectrum of the gold colloid. By subtracting the reference spectrum from the gold colloid spectrum and applying lock-in processing, the absorption spectrum of the gold particles can be specifically obtained. The results are shown in Figure 7a–c. It is noticeable that the sun influenced intensively on the signal quality in Figure 7a,b.

The normalized intensity becomes close to 1.0 in the wavelength range between 526.6 and 531.8 nm, with the peak value (equal to 1.0) at 527.1 nm, which corresponds to the range of highest absorption of gold nanoparticles [49,50]. The normalization constant applied in the graphs in Figure 7c was obtained from the spectrum intensity values after lock-in processing. Considering the normalized mean spectral intensity of the noise of the post-processing experiment was approximately 0.55, it appears that the SNR was approximately 4.4 dB. While the signal quality might not be as good as in the previous experiment, it is still possible to detect the same peak, which shows that SpectroPlot is capable of detecting absorption peak in outdoor environments without luminosity control.

The methodology used in the optical absorption experiment can be extended to other spectroscopy methods, including fluorescence, reflection, polarimetry, etc. Therefore, the methodology opens up new possibilities for exploring spectroscopy techniques in outdoor conditions.

### 3.3. Outdoor Analysis

In order to analyze the impact of solar radiation fluctuations on the detected modulated signal, the setup illustrated in Figure 4d was utilized. The solar radiation intensity is obtained with a Kipp & Zonen^®^ CMP6 pyranometer (Kipp & Zonen, Delftechpark, Delft, The Netherlands) and stored in a data logger while the spectrometer measurement is acquired.

The system’s robustness was evaluated by analyzing the similarity between the obtained curves under various external light fluctuations. The experiment lasted 15 min, and a modulation frequency of 1 Hz was used, resulting in 900 normalized spectra. A Matlab routine was developed to compare each spectrum with the spectrum acquired at the end of the measurement time. This routine calculates the correlation coefficient using the two-dimensional correlation function, *r*, which represents the degree of similarity between two spectra, admitting that *A* is the curve of the optical spectrum at any given time, and *B* is the curve of the optical spectrum at the last instant. The mathematical representation of the correlation function is shown in [52].

(10) $$r\;=\;\frac{\sum_{m}\sum_{n}\left(A_{mn}-\bar{A}\right)\left(B_{mn}-\bar{B}\right)}{\sqrt{\left(\sum_{m}\sum_{n}{\left(A_{mn}-\bar{A}\right)}^{2}\right)\left(\sum_{m}\sum_{n}{\left(B_{mn}-\bar{B}\right)}^{2}\right)}},$$

The subscripts *m* and *n* are used to specify the position of each point in the spectra on the cartesian plane, represented, respectively, as the wavelength and normalized intensity. The mean values of each spectrum are represented as $\bar{A}$ and $\bar{B}$.

**Figure 8:**
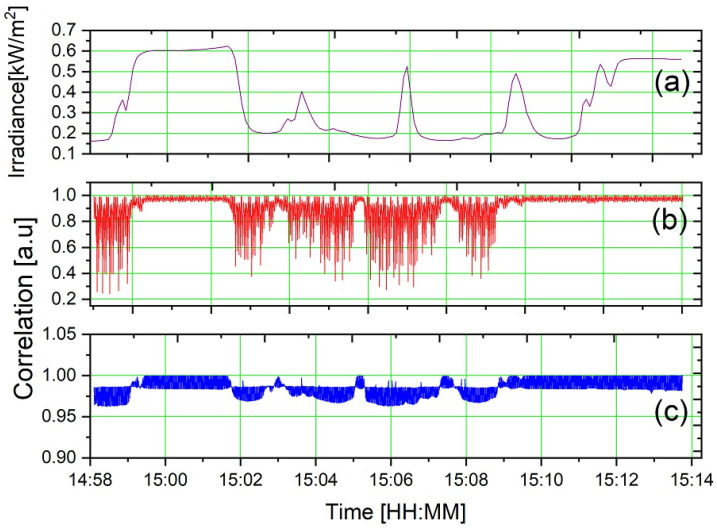
Irradiance of (**a**) gold colloidal solution correlation before (**b**) and after (**c**) lock-in processing.

To validate the method, we conducted several experiments at different time intervals on various days and at different moments during a day. A sample of these experiments is presented in Figure 8a, performed on 7 April 2021 from 14:58 to 15:14, with a solar irradiance of 623 W/m${}^{2}$ and fluctuations of 65%, which are typical values for a cloudy day. The impact of solar radiation fluctuations on the absorption spectrum can be observed in Figure 8b without lock-in processing. As shown in Figure 8c, with lock-in processing, the correlation is practically equal to 1.0 during all the experiments; even with maximum irradiance fluctuations of 65%, the maximum correlation variation is next to 3%, indicating that the proposed methodology allows for performing outdoor spectroscopy experiments.

## 4. Conclusions

The simultaneous lock-in processing in a linear CCD array has been proven to be effective in optical spectroscopy experiments without the need for luminosity control. The method can recover the emission spectrum of a modulated yellow LED (589 nm) with a frequency of 25 Hz, immersed in different surrounding emulated noise (i.e., green led and white-light illumination). The Signal-to-Noise Ratio (SNR) of −70.45 dB demonstrates the robustness of the proposed method.

The absorption spectrum of a gold nanoparticle colloid with a peak around 527 nm can be obtained indoors and outdoors under controlled and uncontrolled external illumination conditions, respectively. From a correlation experiment between consecutive absorption spectra of the nanoparticle subjected to solar radiation, it was possible to establish a correlation close to 1 (one) using the proposed methodology, verifying the capacity of the method to exclude the external solar fluctuations. It is possible to explore the proposed method in other optical techniques, such as diffraction, scattering, fluorescence, microscopy, and polarimetry, for industrial and commercial applications without the need to control external illumination fluctuations.

The processing module can be designed from any CCD spectrometer, and the lock-in processing program can be implemented in any programming language. However, it should be noted that the limitations of the proposed technique are directly related to the acquisition rate of the optical spectra from the CCD spectrometer. The higher this rate, the higher the modulation frequency of the light source and, consequently, the lower influence of lighting conditions. The modulation module can be developed on any microcontroller platform since it is usual for microcontrollers to have a PWM module. In addition to robustness, SpectroPlot also has low computational effort and is aligned with the Industrial Internet of Things-IIoT. It is possible to find portable spectrometers with communication interfaces compatible with these platforms.

## Data Availability

The data presented in this study are available upon request from the corresponding author.
